# Mutations Found in *embCAB*, *embR*, and *ubiA* Genes of Ethambutol-Sensitive and -Resistant *Mycobacterium tuberculosis* Clinical Isolates from China

**DOI:** 10.1155/2015/951706

**Published:** 2015-08-31

**Authors:** Yuhui Xu, Hongyan Jia, Hairong Huang, Zhaogang Sun, Zongde Zhang

**Affiliations:** ^1^Molecular Laboratory, Beijing Chest Hospital, Capital Medical University, 97 Machang Road, Tongzhou District, Beijing 101149, China; ^2^Beijing Key Laboratory for Drug Resistance Tuberculosis Research, Beijing Tuberculosis and Thoracic Tumor Research Institute, 97 Machang Road, Tongzhou District, Beijing 101149, China; ^3^National Tuberculosis Clinical Laboratory, Beijing Chest Hospital, Capital Medical University, 97 Machang Road, Tongzhou District, Beijing 101149, China

## Abstract

To better understand the molecular mechanisms of Ethambutol (EMB) resistance, the mutant hot spot region of five genes (*embB, embA, embC, embR*, and *ubiA*) was amplified and sequenced in 109 EMB-resistant and 153 EMB-susceptible clinical isolates from China. Twenty-seven EMB-susceptible isolates were found to have nonsynonym mutations, 23 of which were in *embB*. The mutations occurred most frequently in *embB* (85.3%, 93) and were seldom in *embC* (2.8%, 3), *embA* (3.7%, 4), *embR* (3.7%, 4), and *ubiA* (8.3%, 9) in EMB-resistant isolates. For the *embB* gene, 63 isolates showed mutations at *embB*306, 20 at *embB*406, nine at *embB*497, and five at *embB*354 in EMB-resistant isolates. In addition, the particular mutants at *embB*406 and *embB*497 indicated both high levels of EMB resistance (MICs > 5 *μ*g/mL) and broad anti-TB drug resistance spectrums. Our data supported the facts that *embB*306 could be used as a marker for EMB resistance with a sensitivity of 57.8% and a specificity of 78.8%.

## 1. Introduction

Ethambutol (EMB) is an antituberculosis drug that is widely used for treating drug resistance, and it is also commonly used for treating multidrug-resistant tuberculosis [1]. The collective results of the EMB drug susceptibility test (DST) of clinical* M. tuberculosis* strains, which has been extensively reviewed in many countries, indicate that many of the strains are resistant to EMB [2–4]. Due to the numerous EMB-resistant strains, the mechanisms underlying EMB resistance, namely, mutations related to EMB target genes, have been both investigated and summarized [4–8].

EMB appears to inhibit arabinosyl transferases encoded by the* embCAB* operon, which is involved in polymerizing arabinose into the arabinan components of arabinogalactan and lipoarabinomannan. The mutations in the* embCAB* operon are responsible for its resistance, especially the “canonical” mutations in codons 306, 406, or 497 of* embB* [4, 9, 10]. Belanger et al. (1996) reported that* embR* modulates the level of arabinosyltransferase activity* in vitro*, which might confer EMB resistance [11].* embR* may control arabinosyltransferase activity in* M. tuberculosis* in a phosphorylation-dependent fashion, acting downstream of the Ser/Thr-kinase PknH [11]. Recently, Safi et al. found that mutations on the gene* ubiA* were associated with high-level resistance and had multiplicative effects with* embB* mutations on minimum inhibitory concentrations (MICs) [4]. The* ubiA* gene encoding 5-phospho-alpha-d-ribose-1-diphosphate:decaprenyl-phosphate 5-phosphoribosyltransferase is known to be essential for the growth of* M. tuberculosis*, and EMB was found to inhibit other steps in arabinan biosynthesis [12]. Although EMB does not directly inhibit* ubiA*,* ubiA* mutations have been shown to increase DPA synthesis, causing the MICs to increase in both a wild-type background and an* embB* codon 306 mutant background [4].

The* embB* mutations that are related to the EMB target genes have been extensively studied, but studies regarding* embA*,* embC*,* embR*, and* ubiA* mutations are lacking. Moreover, less data has been generated on the simultaneous presence of these particular gene mutations in a large amount of clinical isolates. In this study, we sequenced the five genes to find the concomitant existence of the mutations in 109 clinical isolates. This study was an important step towards gaining a full understanding of the molecular mechanisms of EMB resistance and the mutation patterns in clinical isolates from China.

## 2. Materials and Methods

### 2.1. Bacterial Strains and Susceptibility Testing

*M. tuberculosis* H37Rv (ATCC 27294), which was used as the control for the antibiotic susceptibility test, was obtained from the Beijing Bio-Bank of Clinical Resources on Tuberculosis for the isolates. From January 1, 2009, to December 31, 2009, a total of 109 EMB-resistant clinical* M. tuberculosis* isolates were collected from 1,048 isolates. The absolute two-concentration method was conducted twice in order to determine the low or high resistance levels on Lowenstein-Jensen (L-J) slants [13]. The 109 EMB-resistant and 153 randomly selected EMB-susceptible isolates included in this study were also subjected to susceptibility testing for isoniazid (INH), rifampicin (RFP), streptomycin (SM), EMB, ofloxacin (OFX), Capreomycin (CPM), para-aminosalicylic acid sodium (PAS), and amikacin (AMK) by the absolute concentration method [9]. The INH, RFP, OFX, SM, EMB, CPM, PAS, and AMK were purchased from Sigma-Aldrich (Beijing, China). They were dissolved to 100-fold concentrated stock solutions according to the manufacturer's instructions.

### 2.2. DNA Extraction and PCR Amplification

The genomic DNA from the samples was isolated from the mycobacterial cultures using the QIAmp DNA Mini Kit (Qiagen, CA, USA). PCR was performed for various gene loci of* embC*,* embA*,* embR*,* ubiA*, and the enlarged* embB* primers. The primer sets that were used are described in Table 1. The primers were designed based on the H37Rv gene sequence (NC_000962.3) with the Oligo 6.0 software (Wojciech Rychlik Molecular Biology Insights, Inc., CO, USA). Only EMB resistance-determining regions (ERDR) that were amplified in the PCR reactions, including codons 655-988 of the* embC* gene, codons 203-906 and 858-1196 of the* embA* gene, codons 640-1002, 898-1423, and 1405-1747 of the* embB* gene, codons 17-865 of the* ubiA* gene, and codons 24-1160 of the* embR* gene (Table 1), were the ones that had been previously reported. The DNA templates for the PCR products were purified using a QIAquick PCR Cleanup Kit (QIAGEN, CA, USA) as per the manufacturer's instructions and were subjected to DNA sequencing.

### 2.3. Sequencing and Data Analysis

All PCR products that were utilized in this research were sequenced by Sangon Co. Ltd. in China. The sequencing data was assembled by SeqMan Pro (version 7.1, DNAstar Lasergene), and the mutations that were uncovered were identified by comparison with the H37Rv sequences (NC_000962.3) of* embB*,* embA*,* embC*,* embR*, and* ubiA* from the GenBank database (http://www.ncbi.nlm.nih.gov/nuccore/NC_000962.3) using the MegAlign (version 7.1, DNAstar Lasergene). Both the frequency calculations and the association analyses were performed using GraghPad 5 for Windows (GraghPad, Inc., USA).

## 3. Results

### 3.1. Antibiotic Susceptibility Testing

Among the 1,048 isolates collected between January 1, 2009, and December 31, 2009, a total of 109 clinical* M. tuberculosis* isolates were EMB-resistant, of which 67 were MDR-TB isolates, 11 were XDR-TB isolates, 26 were resistant to INH or RFP, and the remaining 5 were resistant to neither INH nor RFP. The results of the drug susceptibility tests are shown in Table 2. The absolute two-concentration, concentration DST results showed that 34 isolates had a high EMB concentration level (MICs ≥ 5), and the remaining 75 EMB-resistant isolates had a low EMB concentration level (2 ≤ MICs < 5).

To further investigate the drug-resistant spectrum, the 109 EMB-resistant isolates and 153 randomly selected EMB-sensitive isolates were also subjected to susceptibility testing for INH, RFP, SM, EMB, OFX, CPM, PAS, and AMK. Results showed that the EMB-resistant isolates were resistant to an average of 3.49 ± 1.59 (mean ± SD) of the eight tested anti-TB drugs, while the EMB-sensitive isolates were resistant to an average of 0.72 ± 0.41 (mean ± SD) of the eight tested anti-TB drugs. The highly EMB-resistant isolates were resistant to an average of 4.58 ± 1.96, whereas the isolates with low EMB resistance were resistant to an average of 2.94 ± 1.02. The 153 EMB-sensitive isolates were resistant to an average of 0.73 ± 0.42 of the eight tested anti-TB drugs.

### 3.2. Mutations in the Tested Genes

Of all the 153 EMB-susceptible isolates, only one or two were found to have nonsynonym mutations in* embC*,* embA*,* embR,* and* ubiA*. Among the 109 EMB-resistant isolates, there were one, four, four, and six isolates with nonsynonym mutations in the* embC*,* embA*,* embR*, and* ubiA*, respectively. The mutation pattern in* embA* included V343L, L105V, and R380P, for EMB-resistant, and V343L, R380P for EMB-susceptible isolates. In* embR*, nonsynonym mutations occurred at P49A, S104N, and P243S in EMB-resistant isolates and at L125S and R230W in EMB-susceptible isolates. Nonsynonym mutations occurred at S244T, I179T, E149D, and A38T in EMB-resistant isolates and none in EMB-susceptible isolates in* ubiA*. Only one nonsynonym mutation was found in* embC* at E305D, which was found in EMB-resistant isolates, and a synonym mutation occurred at E305E, which was found in both EMB-resistant and EMB-susceptible isolates.

The* embB* mutation rate in 109 EMB-resistant* M. tuberculosis* strains was 85.3% (93/109) but was only 15.0% (23/153) in EMB-susceptible strains, of which 17 were at the site of* embB306* (Table 3). Other* embB* mutation patterns were also found at codons 328 (3), 354 (5), 406 (20), and 497 (9) in EMB-resistant isolates and at codons 246 (1), 307 (1), 318 (1), 336 (1), 406 (1), and 439 (1) in 153 EMB-susceptible isolates. Eleven isolates had double* embB* mutations in EMB-resistant isolates. Of these 11, 10 carried the mutation at the site of* embB*306 combined with either* embB*406,* embB*497,* embB*354, or* embB*328.

Mutations at* embB306* were most common, as they were found in both EMB-resistant (63) isolates (Table 2) and EMB-susceptible (17) isolates (Table 3). The wild type codon ATG in* embB*306 changed into GTG, CTG, TTG, ATA, ATT, or ATC, of which GTG was the most frequent (39), followed by ATA (11), CTG (8), TTG (2), ATT (2), and ATC (1) (Table 2).

### 3.3. Correlation between Mutations and Drug Resistance

Mutations at* embB*497,* embB*354, and* embB*328 were found only in EMB-resistant clinical isolates, and they were considered to correlate to EMB resistance. Mutations at* embB*406 and* embB*306 were also found mainly in EMB-resistant isolates, and they were correlated to EMB resistance with an odd ratio (OR) of 50.7 (*P* < 0.001) and 46.5 (*p* < 0.001), respectively.

Of all the 109 EMB-resistant isolates, the percentage of isolates showing high levels of resistance to EMB (MICs > 5 *μ*g/mL) was not significantly dependent on the presence (39.7%, 25/63) or absence (37.5%, 12/32) of the* embB306* mutation (OR = 1.09, *P* = 0.84). The difference was statistically significant in relation to the presence (60%, 12/20) or absence (33.8%, 25/77) of an* embB*406 mutation (OR = 3.12; *P* = 0.02) as well as the presence (77.8%, 7/9) or absence (34.9%, 30/86) of an* embB*497 mutation (OR = 6.53, *P* = 0.01). Mutations at* embB*328 (*P* = 0.78) and* embB*354 (*P* = 0.70) were not found to be correlated to high EMB resistance. Regression analysis could not be performed in this study, as there were so few EMB-sensitive and -resistant isolates with mutations at* embA*,* embC*,* embR,* and* ubiA*.

Of all the 109 EMB-resistant isolates, more than 18 isolates were found to have mutations (including synonym mutations) in at least two of the five tested genes. When mutations occurred in more than two of the five tested genes, high levels of EMB resistance occurred (OR = 6.2; *P* = 0.001); isolates with mutations in two or more of the tested genes were resistant to more anti-TB drugs (5.87 ± 1.60) than those with mutations in only one of the tested genes (3.03 ± 1.37). Some strains with certain mutation patterns showed broad anti-TB drug-resistant spectrums. The average number of resistant anti-TB drugs for the mutant at* embB*306,* embB*328,* embB*354,* embB*406, and* embB*497 was 2.96 ± 1.07, 4.33 ± 1.53, 2.60 ± 0.89, 5.19 ± 1.23, and 5.75 ± 0.88, respectively.

## 4. Discussion

EMB is an important antimycobacterial drug and is recommended to treat tuberculosis as well as opportunistic infections by* M. avium* in patients with acquired immunodeficiency syndrome [14]. However, EMB resistance has been reported frequently in many countries. The traditional views of the mechanisms for EMB resistance mainly focus on the mutations of the* embB* gene, which creates resistance by altering drug-protein interaction. Including the* embCAB* operon, the transcriptional regulators* embR* and* ubiA* have also been associated with EMB-resistant* M. tuberculosis* [15]. In the present study, we sequenced the* embCAB* operon,* embR*, and* ubiA* in 109 EMB-resistant and 153 EMB-sensitive* M. tuberculosis* isolates to find the relationships between the mutations and drug resistance.

Our data supported that mutations in codon* embB* were the predominant mechanism associated with EMB resistance, since 85.3% (93/109) were found to be mutated in EMB-resistant isolates and 15% (23/153) were found to be mutated in EMB-susceptible isolates. High mutation frequencies in* embB* were found at* embB*306 (63),* embB*406 (20),* embB*497 (9), and* embB*354 (5) in EMB-resistant isolates. Other* embB* mutation patterns, such as codons 297, 304, 313, 319, 330, 332, 334, 368, 378, 423, 424, 434, 469, and 508 were not found in this study [4, 16–20]. Previous studies have demonstrated that mutations occur at the* embB* codon 306 in 27% to 87% of EMB-resistant clinical isolates [7, 9, 11, 15, 16, 19, 21–23]. In this study, mutations occurred at the* embB* codon in 55% of the EMB-resistant clinical isolates. Our data supported the facts that* embB*306 could be used as a marker for EMB-resistance with a sensitivity of 57.8% and a specificity of 78.8%. A different frequency of the mutation patterns in the* embB* gene was reported in India. Of all the 52 different positions that were investigated, the most commonly found mutations were located at codon 378 (11), followed by mutations at codons 368 (9), 306 (8), 380 (7), and 406 (6) [21]. This discrepancy may be due to heterogeneity in the methodologies used (e.g., drug susceptibility testing methods) or to the intrinsic molecular variability between isolates from diverse geographical regions.

In this study, all the EMB-resistant isolates with* embB*497 or* embB*406 mutations were MDR-TB, which was consistent with the facts reported by Shi et al. and Srivastava et al. [17, 21]. Moure et al. also reported that the percentage of multidrug resistance among isolates with at least one* embB*406 substitution was significantly higher than that found in the group of isolates without mutations in this codon (100% versus 73.1%, *P* = 0.035). In our report, both higher drug resistance level and broader anti-TB drug spectrum were found in EMB-resistant isolates with* embB497* or* embB406* mutations than in those with* embB306*,* embB328*, or* embB354* mutations.

Mutation in* embB* gene showed lots of patterns in different countries or regions, but quite different for* embC*,* embA,* and* embR*. In this study, the mutations were found mostly in* embB *(85.3%) and less in* ubiA* (8.26%),* embA* (3.7%),* embC* (4.6%), and* embR* (3.7%) in EMB-resistant isolates. In congruence with similar studies conducted in Taiwan, nonsynonymous mutations in* embC* (1),* embA* (4), and* embR* (3) were only rarely encountered in this study [24]. Ramaswamy et al. first reported two nonsynonymous nucleotide substitutions in* embR* resulting in C110W and Q379D replacements [23]. Later, several EMB resistance-associated polymorphisms in* embR* (16/44; 36.3%) were found in India [21]. In mainland China, 2 of 77 EMB-resistant MDR isolates and 4 of 56 EMB-sensitive isolates were found to have mutations in* embC*, and 5 of 74 EMB-resistant MDR-TB and 6 of 54 EMB-sensitive MDR-TB were found to have mutations in* embA* [25]. In New York, USA,* embC* had only 2 EMB resistance-associated nonsynonymous, N394D and R738E, in 75 EMB-resistant samples, and 8 EMB resistance-associated amino acid replacements were identified in* embA* [23]. No mutation was identified in the* embA* gene isolated in India [26], but novel mutations at A254, L251R, T270I, and 297 (11/44) were found in* embC* [21, 27, 28], which we did not find in this study. Some evidence also supported that T270I changed on its own and plays no role in EMB resistance in* embC* [28] and that T270I is not a marker for EMB resistance in the* M. tuberculosis* complex [29]. Mutations in* ubiA* were reported in 19 of 63 that were randomly selected from the World Health Organization Special Programme for Research and Training in Tropical Disease strain bank and in 17 of the 89 isolates from China [30].

To confirm the mutations in the various genes described in the aforementioned literature, allelic exchange experiments were carried out. Safi et al. confirmed that mutations of M306V, M306L, M306I (ATA), and M306I (ATC) all caused EMB resistance (MIC = 4 mg/L) when incorporated into wild-type strains 210 and 5310 [31]. The fold increase in EMB MIC was also investigated for M306V, M306I (ATA), and M306I (ATC) that had been introduced into H37Rv by Starks et al. [26] and Plinke et al. [32]. Safi et al. also looked at the role of common mutations found in clinical strains with high-level EMB resistance at the* embB* 406 and 497 codons [10]. By introducing the point mutation in* embC*, Goude et al. verified that the mutations D294G, M300L, and M300V increased susceptibility to EMB and that mutation M300I had no resistance effect [28]. The introduction of* Rv3806c* mutations into either codon 18, 188, 237, 240, 249, 174, 176, or 175 caused the increase of EMB MIC [4, 30], but not into codon 149 [4]. Newly found mutations at codons 38, 254, 198, and 249 of* Rv3806c* must be studied further.

In this study, no mutations were found at the tested sites in 14 of the total 109 isolates and 17 EMB sensitive isolates were found with the mutations of* embB306*. The discrepancy in drug susceptibility between the phenotype and the genotype was multifactorial. Those factors included the overlapping of the MIC distributions between the wild-type and mutant strains [33], the heteroresistance from the bacterial population [22, 34], the limitation of the current DST [35, 36], and the bacterial itself changes in the cell wall thickness, the efflux pump activity and mutations at other genes not included in this study [25, 38].

## 5. Conclusion

In conclusion, we have demonstrated that mutations were frequently found in the* embB* gene, especially in EMB-resistant* M. tuberculosis* strains. The* embB*306,* embB*497, and* embB*406 mutation patters were ranked as the top three in mutation frequency and were found to be associated with EMB resistance. In addition, the particular mutants at* embB*406 and* embB*497 indicated both high levels of EMB resistance (MICs > 5 *μ*g/mL) and broad anti-TB drug resistance spectrums. The features of EMB resistance revealed in this study will increase our understanding of the distribution and frequency of mutations in* M. tuberculosis* isolates with EMB resistance in TB patients from China.

## Figures and Tables

**Table 1:** Primers employed in this study.

Genes	Primers (5′-3′)	Annealing temperature (°C)	PCR products (bp)
*embC*	F: GATACCCGCTACAGCAGCA	63	334
	R: GGTCGTAGTACCAGCCGAAA		

*embA1*	F: GCCGGCTATGTAGCCAACTA	63	338
	R: GACCGTTCCACCAACACC		

*embA2*	F: GCGCGCTGGACATCTCGAT	68	704
	R: CGCCTCCGTCGTGCCGAAATA		

*embB1*	F: CCGACCACGCTGAAACTGC	63	364
	R: GTAATACCAGCCGAAGGGATCCT		

*embB2*	F: GACGGCTACATCCTGGGCATG	68	525
	R: TGCCGACCAGGCGATGACG		

*embB3*	F: CGTCATCGCCTGGTCGGCAC	64	812
	R: ACATGGTGCCGAAGATGACGC		

*embR*	F: CGCTGATCTGGAACGTGAAT	65	1137
	R: GTAGCGCGACAGTGGAGAAG		

*ubiA*	F: TGACTCAACCTCCGGCAAACC	63	850
	R: GCGCCAGCAGCTGCAATACCC		

**Table 2:** Characteristics of the mutants in* embB, embA, embC, embR,* and* ubiA* within the EMB-resistant isolates.

Types	Locus, nucleotide change, and amino acid change					Number (*n* = 109)	High resistance (*n* = 37)	Resistant spectrum (mean ± SD = 3.49 ± 1.59)	MDR/XDR
	*embB*	*embA*	*embC*	*embR*	*ubiA*				
1	WT	WT	WT	WT	WT	14	0	1.8	6 none, 8 MDR
2	M306V (ATG-GTG)	WT	WT	WT	WT	22	5	1.5	9 none, 13 MDR
3	M306L (ATG-CTG)	WT	WT	WT	WT	8	3	1.9	2 none, 6 MDR
4	M306L (ATG-TTG)	WT	WT	WT	WT	2	1	2.2	1 none, 1 MDR
5	M306I (ATG-ATA)	WT	WT	WT	WT	8	2	3.8	3 none 4 MDR, 1 XDR
6	M306I (ATG-ATT)	WT	WT	WT	WT	1	0	4	1 MDR
7	M306I (ATG-ATT)	WT	WT	WT	E149D (GAA-GAC)	1	1	8	1 XDR
8	M306I (ATG-ATA) G406S (GGC-AGC)	WT	WT	WT	WT	1	1	5	MDR
9	M306I (ATG-ATA) G406D (GGC-GAC)	WT	WT	WT	WT	1	1	4	MDR
10	M306I (ATG-ATA) G406D (GGC-GAC)	WT	WT	462 (c-del)	V49L (GTC-CTC) *P254P (CCG-CCT)*	1	1	4	MDR
11	M306I (ATG-ATC) R354S (AGA-AGC)	V122G (GTG-GGG) V125G (GTG-GGG)	WT	WT	WT	1	1	4	MDR
12	M306V (ATG-GTG)	V343L (GTG-TTG)	WT	WT	WT	1	0	6	MDR
13	M306V (ATG-GTG)	WT	E305D (GAG-GAC)	WT	WT	1	1	7	XDR
14	M306V (ATG-GTG)	WT	WT	G84G (GGT-GGG) S104N (AGC-AAC)	A38T (GCC-ACC)	1	1	5	MDR
15	M306V (ATG-GTG)	WT	WT	WT	L198L (CTG-CTC)	1	1	8	XDR
16	M306V (ATG-GTG)	WT	WT	WT	*I206I (ATC-ATT)*	1	0	3	None
17	M306V (ATG-GTG)	L105V (CTG-GTG)	WT	WT	WT	1	0	7	XDR
18	M306V (ATG-GTG)	R380P (CGT-CCT)	WT	WT	WT	1	0	3	MDR
19	M306V (ATG-GTG) D328H (GAT-CAT)	WT	WT	WT	WT	1	0	3	MDR
20	M306V (ATG-GTG) D328G (GAT-GGT)	WT	WT	WT	WT	1	1	6	MDR
21	M306V (ATG-GTG) G406P (GGC-CCG) *A439A (GCA-GCG)*	WT	WT	WT	E149D (GAA-GAC)	1	1	7	XDR
22	M306V (ATG-GTG) G406P (GGC-CCG) *A439A (GCA-GCG)*	WT	WT	WT	*I206I (ATC-ATT)*	1	1	6	MDR
23	M306V (ATG-GTG) G406A (GGC-GCC)	WT	WT	WT	WT	1	1	3	MDR
24	M306V (ATG-GTG) Q497R (CAG-CGG)	WT	*E305E (GAG-GAA)*	WT	WT	1	1	4	MDR
25	M306V (ATG-GTG) *D531D (GAC-GAT)*	WT	WT	WT	WT	4	1	5.5	2 none, 2 MDR
26	D328H (GAT-CAT)	WT	WT	WT	WT	1	0	4	MDR
27	R354S (AGA-AGC)	WT	WT	WT	WT	3	0	2	1 none, 2 MDR
28	R354S (AGA-AGC) *D531D (GAC-GAT)*	WT	WT	WT	WT	1	0	3	None
29	G406P (GGC-CCG)	WT	WT	WT	WT	1	1	7	XDR
30	G406P (GGC-CCG) *A439A (GCA-GCG)*	WT	WT	WT	WT	2	0	5.5	MDR
31	G406D (GGC-GAC)	WT	WT	WT	WT	4	2	5	3 MDR, 1 XDR
32	G406A (GGC-GCC)	WT	WT	WT	WT	6	2	5.5	5 MDR, 1 XDR
33	G406A (GGC-GCC)	WT	*E305E (GAG-GAA)*	WT	WT	1	0	5	MDR
34	G406C (GGC-TGC)	WT	WT	WT	WT	1	1	4	MDR
35	*A439A (GCA-GCG)*	WT	WT	WT	WT	2	0	3.5	2 none
36	Q497R (CAG-CGG)	WT	WT	WT	WT	5	3	5.4	2 none, 2 MDR, 1 XDR
37	Q497K (CAG-AAG)	WT	WT	WT	WT	1	1	6	MDR
38	Q497R (CAG-CGG) T496N (ACC-AAC)	WT	WT	WT	I179T (ATC-ACC)	1	1	7	XDR
39	Q497R (CAG-CGG)	WT	WT	P49A (CCC-GCC)	WT	1	1	6	None
40	WT	WT	WT	P49A (CCC-GCC) P243S (CCC-TCC)	WT	1	0	4	MDR
41	WT	WT	WT	WT	S244T (AGC-ACC)	1	0	5	MDR

WT: wild type; MDR: multidrug resistance; XDR: extensively drug resistance.

**Table 3:** Mutants in* embB, embA, embC, embR*, and *ubiA* within the EMB-sensitive isolates.

Types	Locus, nucleotide change, and amino acid change					Number (*n* = 153)
	*embB*	*embA*	*embC*	*embR*	*ubiA*	
1	WT	WT	WT	WT	WT	124
2	M306V (ATG-GTG)	WT	WT	WT	WT	11
3	M306L (ATG-CTG)	WT	WT	WT	WT	4
4	M306I (ATG-ATA)	WT	WT	WT	WT	1
5	M306I (ATG-ATT)	WT	WT	WT	WT	1
6	G406P (GGC-CCG)	WT	WT	WT	WT	1
7	G246R (GGC-CGC)	WT	WT	WT	WT	1
8	A307G (GCC-GGC)	WT	WT	WT	WT	1
9	N318S (AAC-AGC)	WT	WT	WT	WT	1
10	A439A (GCA-GCG)	WT	WT	WT	WT	1
11	L336P (CTG-CCG)	WT	WT	WT	WT	1
12	WT	V343L (GTG-TTG)	WT	WT	WT	1
13	WT	R380P (CGT-CCT)	WT	WT	WT	1
14	WT	WT	*E305E (GAG-GAA)*	WT	WT	1
15	WT	WT	WT	L125S (TTG-TCG)	WT	1
16	M306V (ATG-GTG)	WT	WT	R230W (CGT-TGG)	WT	1
17	WT	WT	WT	WT	*I206I (ATC-ATT)*	1
